# Fractal and Entropy Analysis of the Dow Jones Index Using Multidimensional Scaling

**DOI:** 10.3390/e22101138

**Published:** 2020-10-08

**Authors:** José A. Tenreiro Machado

**Affiliations:** Department of Electrical Engineering, Institute of Engineering, Polytechnic Institute of Porto, 4249-015 Porto, Portugal; jtm@isep.ipp.pt; Tel.: +351-228340500

**Keywords:** multidimensional scaling, fractals, fractional calculus, financial indices, entropy, Dow Jones, complex systems

## Abstract

Financial time series have a fractal nature that poses challenges for their dynamical characterization. The Dow Jones Industrial Average (DJIA) is one of the most influential financial indices, and due to its importance, it is adopted as a test bed for this study. The paper explores an alternative strategy to the standard time analysis, by joining the multidimensional scaling (MDS) computational tool and the concepts of distance, entropy, fractal dimension, and fractional calculus. First, several distances are considered to measure the similarities between objects under study and to yield proper input information to the MDS. Then, the MDS constructs a representation based on the similarity of the objects, where time can be viewed as a parametric variable. The resulting plots show a complex structure that is further analyzed with the Shannon entropy and fractal dimension. In a final step, a deeper and more detailed assessment is achieved by associating the concepts of fractional calculus and entropy. Indeed, the fractional-order entropy highlights the results obtained by the other tools, namely that the DJIA fractal nature is visible at different time scales with a fractional order memory that permeates the time series.

## 1. Introduction

The Dow Jones Industrial Average (DJIA), or Dow Jones, is a stock market index that reflects the stock performance of 30 relevant companies included in the U.S. stock exchanges. The DJIA is the second-oldest among the U.S. market indices and started on 26 May 1896. The DJIA is the best-known index in finance and is considered a key benchmark for assessing the global business trend in the world.

The financial time series reflect intricate effects between a variety of agents coming from economic and social processes, geophysical phenomena, health crisis, and political strategies [1,2,3,4]. At present, we find all sorts of financial indices for capturing the dynamics of markets and stock exchange institutions. In general, all have a fractal nature with variations that are difficult to predict [5,6,7,8,9,10,11,12,13]. A number of techniques have been proposed to investigate the financial indices and to unravel the embedded complex dynamics [14,15,16,17,18]. Such studies adopt the underlying concept of linear time flow and consider that the fractal nature of the index is intrinsic to its own artificial nature.

This paper studies the interplay between the DJIA values and the time flow. The present day standard assumption is that time is a continuous linear succession of events often called the “arrow of time”. We must clarify that (i) the nature of the time variable, either continuous or discrete, either with a constant rhythm of variation or not, is simply under the light of the financial index, so that we are independent of the classical laws of physics, (ii) merely the DJIA is adopted since other financial indices reveal the same type of behavior, but are limited to much shorter time series, and (iii) no financial foreseeing is intended. Therefore, the Gedankenexperiment in the follow-up addresses the controversy about the texture of time [19,20,21,22], but just in the limited scope of financial indices.

For this purpose, the concepts of multidimensional scaling (MDS), fractional dimension, entropy, and fractional calculus are brought up as useful tools to tackle complex systems. MDS is a computational tool for visualizing the level of similarity between items of a dataset. The MDS translates information regarding the pairwise distances among a set of items into a configuration of representative points of an abstract Cartesian space [23,24,25,26,27,28,29]. Mandelbrot coined the word “fractal” [30,31] for complex objects that are self-similar across different scales. Fractals can be characterized by the so-called fractal dimension, which may be seen as quantifying complexity [32,33,34]. Information theory was introduced by Claude Shannon [35] and has as the primary concept the information content of a given event, which is a decreasing function of its probability [36,37,38,39]. The entropy of a random variable is the average value of information and has been proven to be a valuable tool for assessing complex phenomena [40,41,42]. Fractional calculus (FC) is the branch of mathematical analysis that generalizes differentiation and integration to real or complex orders [43,44,45,46,47,48]. The topic was raised by Gottfried Leibniz in 1695 and remained an exotic field until the Twentieth Century. In the last few decades, FC became a popular tool for analyzing phenomena with long-range memory and non-locality [49,50,51,52,53,54,55,56,57].

The association of these mathematical and computational tools yields relevant viewpoints when analyzing financial indices [7,8,9,11,58,59,60,61].

Bearing these ideas in mind, this paper is organized as follows. Section 2 introduces the dataset and methods and develops some initial experiments using MDS. Section 3 explores the use of fractal and entropy analysis of the MDS loci. Finally, Section 4 draws the main conclusions.

## 2. Dataset and Methods

### 2.1. The DJIA Dataset

The dataset consists of the daily close values of the DJIA from 28 December 1959, up to 1 September 2020, corresponding to a time series of T= 15,832 days, covering approximately half a century. Each week consists of 5 working days, and some missing data due to special events were estimated by means of linear interpolation between adjacent values.

We assess the dynamics of the DJIA by comparing its values x(t) for a given time window of $t_{w}$ days. Therefore, the *i*th vector of DJIA values consists of $\xi_{i}=\left[x\left(1\right),\ldots ,x\left(t_{w}\right)\right]$, where days “1” and “$t_{w}$” denote the start and end time instants in the time window. Hereafter, for simplicity, we consider consecutive disjoint time windows, and a number of experiments with $t_{w}$ having values multiples of 5 days. Therefore, the total number of time windows (and vectors) is $N_{w}=\left\lfloor \frac{T}{t_{w}}\right\rfloor$, where $\left\lfloor \cdot \right\rfloor$ denotes the floor function, which gives as the output the greatest integer less than or equal to the input value.

**Figure 1:**
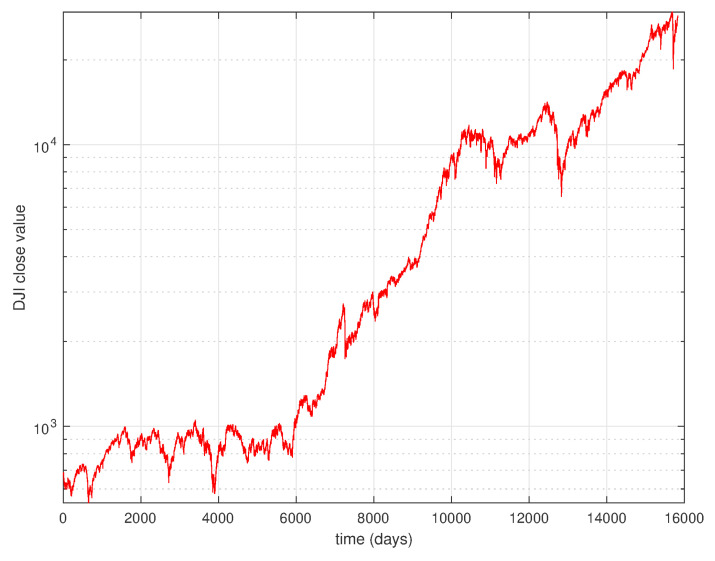
Daily close values of the DJIA from 28 December 1959, up to 1 September 2020.

**Figure 2:**
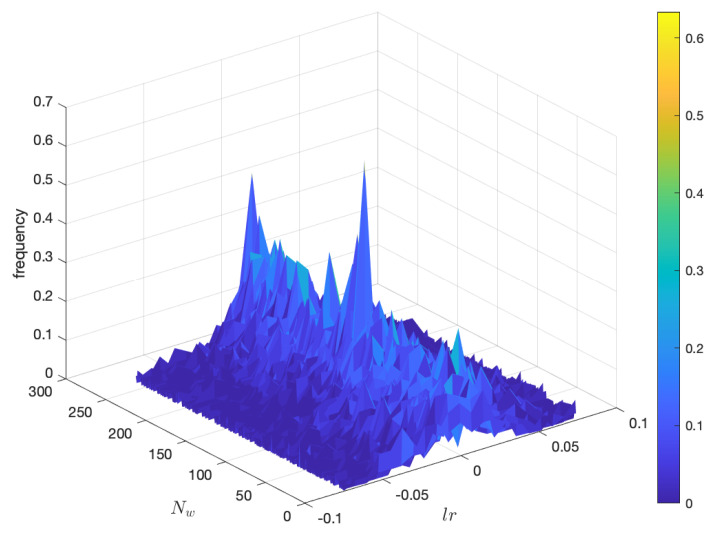
Histogram of the logarithm of the returns of the DJIA from 28 December 1959, up to 1 September 2020, for time windows of $t_{w}=60$ days.

The evolution of the DJIA in time reveals a fractal nature as represented in Figure 1. If we calculate the histogram of the logarithm of the returns, that is of $lr=\ln\left(\frac{x(t+1)}{x(t)}\right)$, we verify a sustained noisy behavior and fat tails in the statistical distribution as depicted in Figure 2 for time windows of $t_{w}=60$ days.

### 2.2. Distances

The DJIA dynamics is studied indirectly through the MDS by comparing the vectors $\left(\xi_{i}\left(1\right),\ldots ,\xi_{i}\left(t_{w}\right)\right)$, $i=1,\ldots ,N_{w}$, $t=1,\ldots ,t_{w}$, and analyzing the properties of the resulting plot in the perspective of entropy and fractal dimension. This approach requires the definition of an appropriate distance [62]. A function d:A×A→R on a set A is a “distance” when, for the items $\xi_{i},\xi_{j},\xi_{k}\in A$, it satisfies the conditions (i) $d(\xi_{i},\xi_{j})\geq 0$ (non-negativity), (ii) $d(\xi_{i},\xi_{j})=0$ (identity of indiscernibles) if and only if $\xi_{i}=\xi_{j}$, (iii) $d\left(\xi_{i},\xi_{j}\right)=d\left(\xi_{j},\xi_{i}\right)$ (symmetry), and (iv) $d\left(\xi_{i},\xi_{k}\right)\leq d\left(\xi_{i},\xi_{j}\right)+d\left(\xi_{j},\xi_{k}\right)$ (triangle inequality). If the three conditions are followed, then the function is a “metric” and together with A yields a “metric space”. Obviously, these conditions still allow a considerable freedom, and we find in the literature a plethora of possible metrics each with its own pros and cons. In practice, users adopt one or more distances if they capture adequately the characteristics of the items under assessment. Therefore, we start by considering a test bench of 10 distinct indices, namely the Manhattan, Euclidean, Tchebychev, Lorentzian, Sørensen, Canberra, Clark, divergence, angular, and Jaccard distances (denoted as {Ma, Eu, Tc, Lo, So, Ca, Cl, Dv, Ac, Ja}), given by [63]:

(1a) $$\begin{array}{cc} d_{i,j}^{Ma} & =\sum_{t=1}^{t_{w}}\left|\xi_{i}\left(t\right)-\xi_{j}\left(t\right)\right|, \end{array}$$

(1b) $$\begin{array}{cc} d_{i,j}^{Eu} & =\sqrt{\sum_{t=1}^{t_{w}}{\left(\xi_{i}\left(t\right)-\xi_{j}\left(t\right)\right)}^{2}}, \end{array}$$

(1c) $$\begin{array}{cc} d_{i,j}^{Tc} & =\max_{t}\left(\xi_{i}\left(t\right)-\xi_{j}\left(t\right)\right), \end{array}$$

(1d) $$\begin{array}{cc} d_{i,j}^{Lo} & =\sum_{t=1}^{t_{w}}\log\left(1+\left|\xi_{i}\left(t\right)-\xi_{j}\left(t\right)\right|\right), \end{array}$$

(1e) $$\begin{array}{cc} d_{i,j}^{So} & =\frac{\sum_{t=1}^{t_{w}}\left|\xi_{i}\left(t\right)-\xi_{j}\left(t\right)\right|}{\sum_{t=1}^{t_{w}}\left(\left|\xi_{i}\left(t\right)\right|+\left|\xi_{j}\left(t\right)\right|\right)}, \end{array}$$

(1f) $$\begin{array}{cc} d_{i,j}^{Ca} & =\sum_{t=1}^{t_{w}}\frac{\left|\xi_{i}\left(t\right)-\xi_{j}\left(t\right)\right|}{\left|\xi_{i}\left(t\right)\right|+\left|\xi_{j}\left(t\right)\right|}, \end{array}$$

(1g) $$\begin{array}{cc} d_{i,j}^{Cl} & =\sqrt{\sum_{t=1}^{t_{w}}{\left(\frac{\left|\xi_{i}\left(t\right)-\xi_{j}\left(t\right)\right|}{\left|\xi_{i}\left(t\right)\right|+\left|\xi_{j}\left(t\right)\right|}\right)}^{2}}, \end{array}$$

(1h) $$\begin{array}{cc} d_{i,j}^{Dv} & =\sum_{t=1}^{t_{w}}\frac{{\left(\xi_{i}\left(t\right)-\xi_{j}\left(t\right)\right)}^{2}}{{\left(\left|\xi_{i}\left(t\right)\right|+\left|\xi_{j}\left(t\right)\right|\right)}^{2}}, \end{array}$$

(1i) $$\begin{array}{cc} d_{i,j}^{Ac} & =\arccos\left(r_{ij}\right),\;r_{ij}=\frac{\sum_{t=1}^{t_{w}}\xi_{i}\left(t\right)\xi_{j}\left(t\right)}{\sqrt{\sum_{t=1}^{t_{w}}\xi_{i}^{2}\left(t\right)\sum_{t=1}^{t_{w}}\xi_{j}^{2}\left(t\right)}}, \end{array}$$

(1j) $$\begin{array}{cc} d_{i,j}^{Ja} & =\frac{\sum_{t=1}^{t_{w}}{\left(\xi_{i}\left(t\right)-\xi_{j}\left(t\right)\right)}^{2}}{\sum_{t=1}^{t_{w}}\xi_{i}^{2}\left(t\right)+\sum_{t=1}^{t_{w}}\xi_{j}^{2}\left(t\right)-\sum_{t=1}^{t_{w}}\xi_{i}\left(t\right)\xi_{j}\left(t\right)}, \end{array}$$

where $\xi_{i}$ and $\xi_{j}$, $i,j=1,\ldots ,N_{w}$, are the *i*th and *j*th vectors of the DJIA time series, each of dimension $t_{w}$. The Manhattan, Euclidean, and Tchebychev distances are particular cases of the Minkowski distance $d_{i,j}^{Mi}={\left(\sum_{t=1}^{t_{w}}{\left|\xi_{i}\left(t\right)-\xi_{j}\left(t\right)\right|}^{q}\right)}^{\frac{1}{q}}$, namely for q=1, q=2 and q→∞, respectively. The Lorentzian distance applies the natural logarithm to the absolute difference with 1 added to guarantee the non-negativity property and to eschew the log of zero. We find in the literature several distinct versions of the Sørensen distance, eventually with other names, and representing a statistic used for comparing the similarity between two samples. The Canberra and Clark distances are weighted versions of the Manhattan and Euclidean distances. These expressions replace $\left|\xi_{i}\left(t\right)-\xi_{j}\left(t\right)\right|$ by $\left|\xi \left(t\right)-\xi_{j}\left(t\right)\right|\;/\;\left(\left|\xi_{i}\left(t\right)\right|+\left|\xi_{j}\left(t\right)\right|\right)$ and are sensitive to small changes near zero. The angular cosine distance follows the cosine similarity $r_{ij}$ that comes from the inner product of two vectors, $\xi_{i}\cdot \xi_{j}$. The angular cosine distance $d_{i,j}^{Ac}$ gives the angle between the vectors $\xi_{i}$ and $\xi_{j}$. The Jaccard distance is the ratio of the size of the symmetric difference to the union of two sets.

### 2.3. The MDS Loci

Once having defined the metric for comparing the vectors, the MDS requires the construction of a matrix $D=\left[d_{i,j}\right]$ of item-to-item distances. In our case, “item” corresponds to a $t_{w}$-dim vectors. Therefore, the square matrix D is symmetric, with the main diagonal of zeros and dimension $N_{w}\times N_{w}$ equal to the number of items. The MDS computational algorithm tries to plot the items in a low-dimensional space so that users can easily analyze possible relationships that are difficult to unravel in a high number of dimensions. In other words, the MDS performs a dimension reduction and plots items in a $p<N_{w}$ dimensional space, by estimating a matrix $\hat{D}=\left[{\hat{d}}_{i,j}\right]$, corresponding to the *p*-dim items ${\hat{x}}_{i}$, so that the distances, ${\hat{d}}_{i,j}$, mimic the original ones, $d_{i,j}$.

The classical MDS can perform the optimization procedure based on a variety of loss functions, often called “strain”, that are a form of minimizing the residual sum of squares. The metric MDS generalizes the optimization procedure called “stress”, $S_{D}$, such as:

(2) $$S_{D}\left(\xi_{1},\ldots ,\xi \right)={\left[\sum_{i,j}{\left({\hat{d}}_{i,j}-d_{i,j}\right)}^{2}\right]}^{\frac{1}{2}},$$

or:

(3) $$S_{D}\left(\xi_{1},\ldots ,\xi \right)={\left[\frac{\sum_{i,j}{\left({\hat{d}}_{i,j}-d_{i,j}\right)}^{2}}{\sum_{i,j}d_{i,j}^{2}}\right]}^{\frac{1}{2}},$$

where $d_{i,j}=\left|\xi_{i}-\xi_{j}\right|$, $i,j=1,\ldots ,N_{w}$.

The generalized MDS is an extension of metric formulation, so that the target space is an arbitrary smooth non-Euclidean space.

Once having obtained the MDS estimate coordinates of the objects ${\hat{x}}_{i}$, the user can decide the dimension *p* for visualization. Usually, the values p=2 and p=3 are selected since they allow a direct representation. Moreover, the quality of the MDS approximation can be assessed by means of the Sheppard and stress charts. The Sheppard diagram plots ${\hat{d}}_{i,j}$ vs. $d_{i,j}$. If the points follow a straight/curved line, this means a linear/non-linear relationship, but in both cases, the smaller the scatter, the better the approximation is. A second assessment tool consists of the plot of $S_{D}$ vs. *p*. Usually, the curve is monotonic decreasing with a large diminishing at first and a slow variation afterwards.

Since the MDS locus results from relative information (i.e., the distances), the coordinates usually do not have some physical meaning, and the user can rotate, shift, or magnify the representation to have a better view. Moreover, distinct distances lead to different plots that are correct from the mathematical and computational viewpoints, but that reflect distinct characteristics of the dataset. Therefore, it is up to the user to choose one or more distances that better highlight the aspects of the dataset under study.

Often, it is recommended to pre-process the data before calculating the distances in order to reduce the sensitivity to some details such as different units or a high variation of numerical values. In the case of the DJIA, two data pre-processing schemes (also called normalizing, or data transformation), $P_{1}$ and $P_{2}$, are considered: (i) subtracting the arithmetic average and dividing by the standard variation, that is by calculating $\left\{P_{1}:\;x\left(t\right)\leftarrow \frac{x(t)-\mu }{\sigma }\right\}$, where $\mu =\frac{1}{T}\sum_{t=1}^{T}x\left(t\right)$ and $\sigma =\sqrt{\frac{1}{T-1}\sum_{t=1}^{T}{\left(x(t)-\mu \right)}^{2}}$, and (ii) by applying a logarithm so that $\left\{P_{2}:\;x\left(t\right)\leftarrow \mathrm{lg}\left(x\left(t\right)\right)\right\}$. The linear transformation $P_{1}$ is often adopted in statistics and signal processing [64,65,66,67,68], while the non-linear transformation $P_{2}$ can be adopted with signals revealing an exponential-like evolution [69,70,71,72,73]. Of course, other data transformations could be envisaged, but these two are commonly adopted. Therefore, the main question concerning this issue is to understand to what extend the pre-processing influences the final results.

#### 2.3.1. Data Pre-Processing Using $P_{1}$

**Figure 3:**
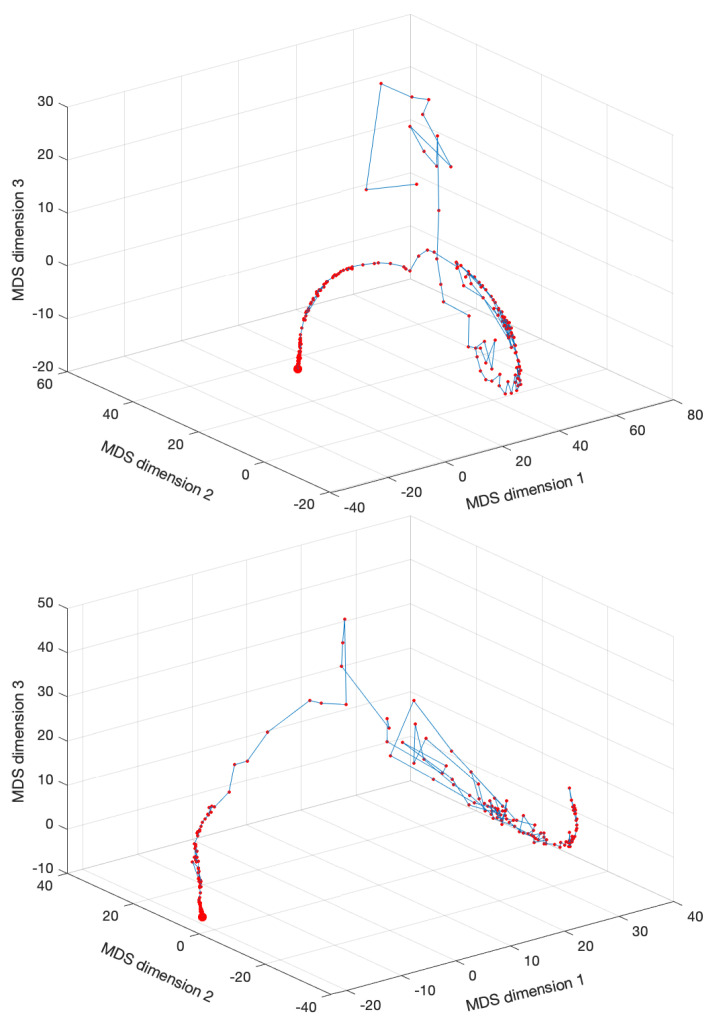
The multidimensional scaling (MDS) locus, ${\hat{x}}_{i}$, of the DJIA dataset for p=3 and $t_{w}=60$ days ($N_{w}=263$), with pre-processing $P_{1}$ and using the Lorentzian (1d) and Canberra (1f) distances.

**Figure 4:**
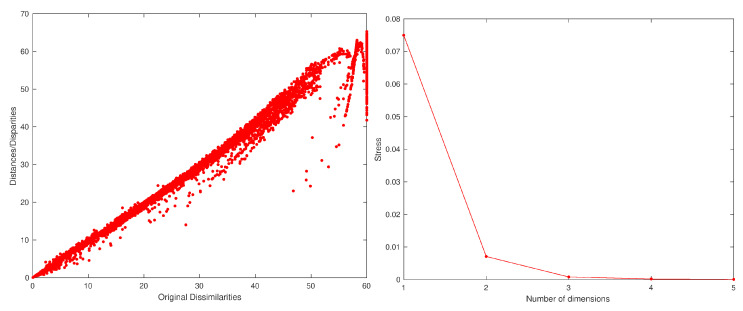
The Sheppard diagram, ${\hat{d}}_{i,j}$ vs. $d_{i,j}$, for p=3, and stress plot, $S_{D}$ vs. *p*, of the DJIA dataset with $t_{w}=60$ days, with pre-processing $P_{1}$ and using the Canberra distance (1f).

Figure 3 shows the MDS locus for p=3 and $t_{w}=60$ days, with pre-processing $P_{1}$ and using the Lorentzian and Canberra distances, $d_{i,j}^{Lo}$ and $d_{i,j}^{Ca}$. The larger circle represents the first vector, and the lines connect two consecutive dots (representing the vectors from two consecutive time windows). The lines are included simply for auxiliary purposes and for highlighting the discontinuities. The MATLAB nonclassical multidimensional scaling algorithm mdscale and the Sammon’s nonlinear mapping criterion sammon were used. Figure 4 illustrates the corresponding Sheppard and stress diagrams for the Canberra distance (1f). For the sake of parsimony, the other charts are not represented.

We verify that the MDS loci exhibit segments where we have an almost continuous evolution and others with strong discontinuities. The first segments portray relatively smooth dynamics, while the second ones represent dramatic variations, in the perspective of the adopted distance and visualization technique. These dynamical effects are not read in the same way as with the standard time representations. Moreover, their visualization varies according to the type of distance adopted to construct the matrix D. This should be expected, since it is well known that each distance highlights a specific set of properties embedded in the original time series and that the selection of one of more distances has to be performed on a case-by-case basis, before deciding those more adapted to the dataset.

**Figure 5:**
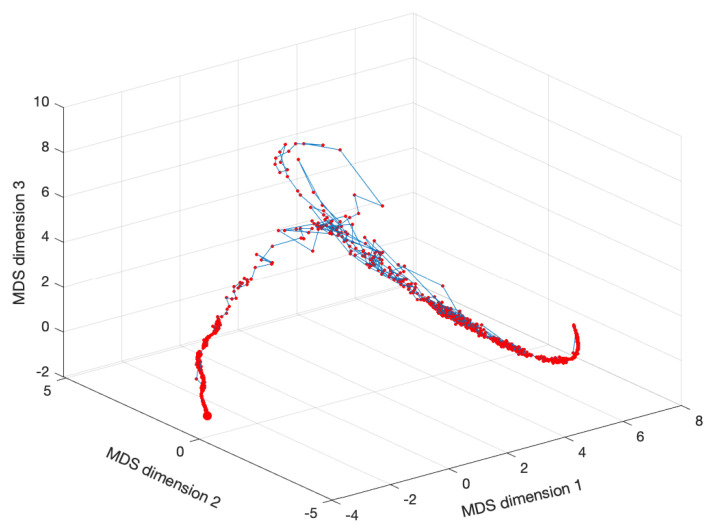
The MDS locus, $\hat{x_{i}}$, of the DJIA dataset for p=3 and $t_{w}=10$ days ($N_{w}=1583$), with pre-processing $P_{1}$ and using the Canberra distance (1e).

Another relevant topic is the effect of the time window $t_{w}$ on the results. In other words, we can ask how the dimension of the vector $\xi_{i}$, $i=1,\ldots ,N_{w}$, capturing the DJIA time dynamics, influences the MDS representation. For example, Figure 5 shows the MDS locus for p=3, $t_{w}=10$ days ($N_{w}=1583$), and the Canberra distance (1e).

#### 2.3.2. Data Pre-Processing Using $P_{2}$

**Figure 6:**
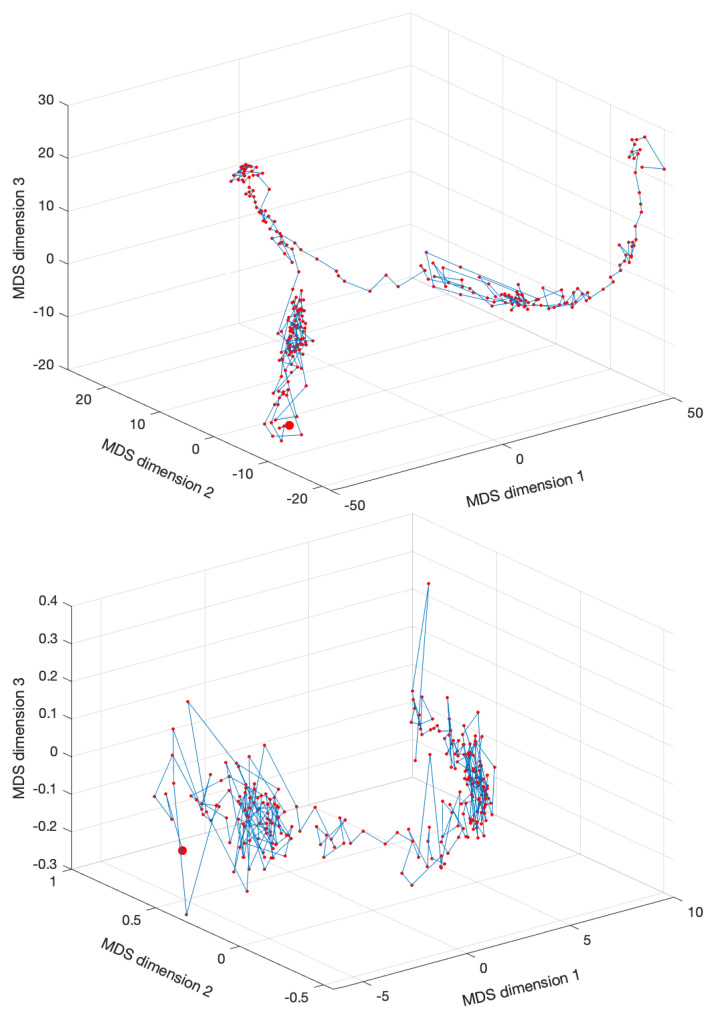
The MDS locus, ${\hat{x}}_{i}$, of the DJIA dataset for p=3 and $t_{w}=60$ days ($N_{w}=263$), with pre-processing $P_{2}$ and using the Lorentzian (1d) and Canberra (1f) distances.

**Figure 7:**
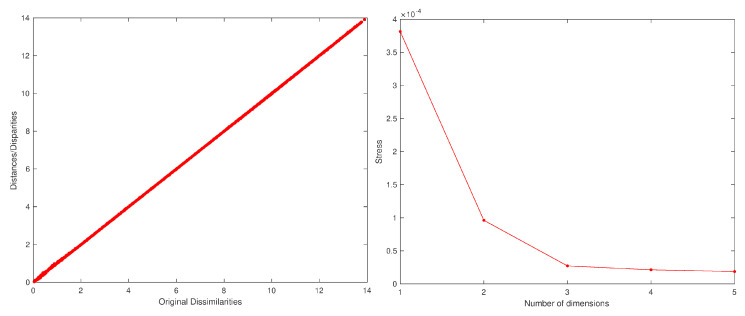
The Sheppard diagram, ${\hat{d}}_{i,j}$ vs. $d_{i,j}$, for p=3, and the stress plot, $S_{D}$ vs. *p*, of the DJIA dataset with $t_{w}=60$ days, with pre-processing $P_{2}$ and using the Canberra distance (1f).

Figure 6 shows the MDS locus for p=3 and $t_{w}=60$ days, with pre-processing $P_{2}$ and using the Lorentzian and Canberra distances, $d_{i,j}^{Lo}$ and $d_{i,j}^{Ca}$. Figure 7 depicts the Sheppard and stress diagrams for the Canberra distance (1f).

**Figure 8:**
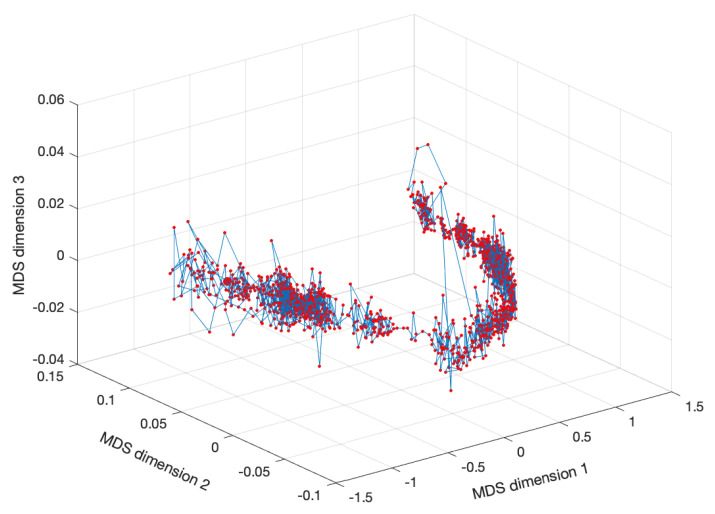
The MDS locus, $\hat{x_{i}}$, of the DJIA dataset for p=3 and $t_{w}=10$ days ($N_{w}=1583$), with pre-processing $P_{2}$ and using the Canberra distance (1e).

We can also check the effect of the time window $t_{w}$. Figure 8 shows the MDS locus for p=3, $t_{w}=10$ days ($N_{w}=1583$), and the Canberra distance (1e) revealing, again, a slightly diminishing of the volatility.

As in the previous sub-section, we observe that the MDS plots reveal some segments almost with a continuous evolution and some with discontinuities. Furthermore, as before, increasing $t_{w}$ reduces the volatility in the MDS representations. These results, with regions of smooth variation, interspersed with abrupt changes, were already noticed since they reflect relativistic time effects [74,75]. Such dynamics was interpreted as a portrait of the fundamental non-smooth nature of the flow of the time variable underlying the DJIA evolution. Nonetheless, we are still far from a comprehensive understanding of the MDS loci, and we need to design additional tools to extract additional conclusions.

## 3. Fractal, Entropy, and Fractional Analysis

We consider the fractal dimension and entropy measures for analyzing the 3-dim portraits produced by the MDS.

The fractal dimension, $f_{d}$, characterizes the fractal pattern of a given object by quantifying the ratio of the change in detail to the change in scale. Several types of fractal dimension can be found in the literature. In our case, $f_{d}$ is calculated by means of the box counting method as the exponent of a power law $N\left(\epsilon \right)=a\epsilon^{-f_{d}}$, where *a* is a parameter that depends on the shape and size of the object, and *N* and ϵ stand for the number of boxes required to capture the object and the size (or scale) of the box, respectively. Therefore, $f_{d}$ can be estimated as:

(4) $$f_{d}=-\lim_{\epsilon \to 0}\frac{\ln\left(N\left(\epsilon \right)\right)}{\ln\left(\epsilon \right)}.$$

The entropy of a random variable is the average level of “information” of the corresponding probability distribution. The key cornerstone of the Shannon theory consists of the information content, which for an event having probability of occurrence $p_{i}$, is given by:

(5) $$I\left(p_{i}\right)=-\ln p_{i}.$$

For a 3-dim random variable $\left(X,Y,Z\right)$ with probability distribution $p_{XYZ}$, the Shannon entropy, $H_{XYZ}$, is given by:

(6) $$H_{XYZ}=-\sum_{X}\sum_{Y}\sum_{Z}p_{XYZ}\ln\left(p_{XYZ}\right),$$

where $-\ln\left(p_{XYZ}\right)$ is the information for the event with probability $p_{XYZ}$.

The concept of entropy can be generalized in the scope of fractional calculus [76,77,78,79,80,81,82,83,84,85,86]. This approach gives more freedom to adapt the entropy measure to the phenomenon under study by adjusting the fractional order. The information and entropy of order α∈R are given by [77,87]:

(7) $$I_{\alpha }\left(p_{i}\right)=D^{\alpha }I\left(p_{i}\right)=-\frac{p_{i}^{-\alpha }}{\Gamma \left(\alpha +1\right)}\left[\ln p_{i}+\psi \left(1\right)-\psi \left(1-\alpha \right)\right]$$

(8) $$H_{XYZ}^{\alpha }=\sum_{i}\left\{-\frac{p_{i}^{-\alpha }}{\Gamma \left(\alpha +1\right)}\left[\ln p_{i}+\psi \left(1\right)-\psi \left(1-\alpha \right)\right]\right\}p_{i}$$

where $\Gamma \left(\cdot \right)$ and $\psi \left(\cdot \right)$ represent the gamma and digamma functions.

The parameter α gives an extra degree of freedom to adapt the sensitivity of the entropy calculation of each specific data series.

In an algorithmic perspective, these measures require the adoption of some grid (or box) for capturing and counting the objects, the main difference being that the fractal dimension just considers a Boolean perspective of “1” and “0”, that is the box is either full or empty, while the entropy considers the number of counts in each box.

In the follow-up, a 3-dim grid defined between the minimum and maximum values obtained for each axis of the MDS locus is considered. For the fractal dimension, we obtain $f_{d}$ by the slope of $N\left(\epsilon \right)$ versus ϵ for 10 decreasing values of the box sizes. In the case of the entropy, we calculate $H_{XYZ}$ when adopting 20 bins for each MDS axis. The auxiliary lines connecting the object (i.e., the points) are not considered for the calculations.

**Figure 9:**
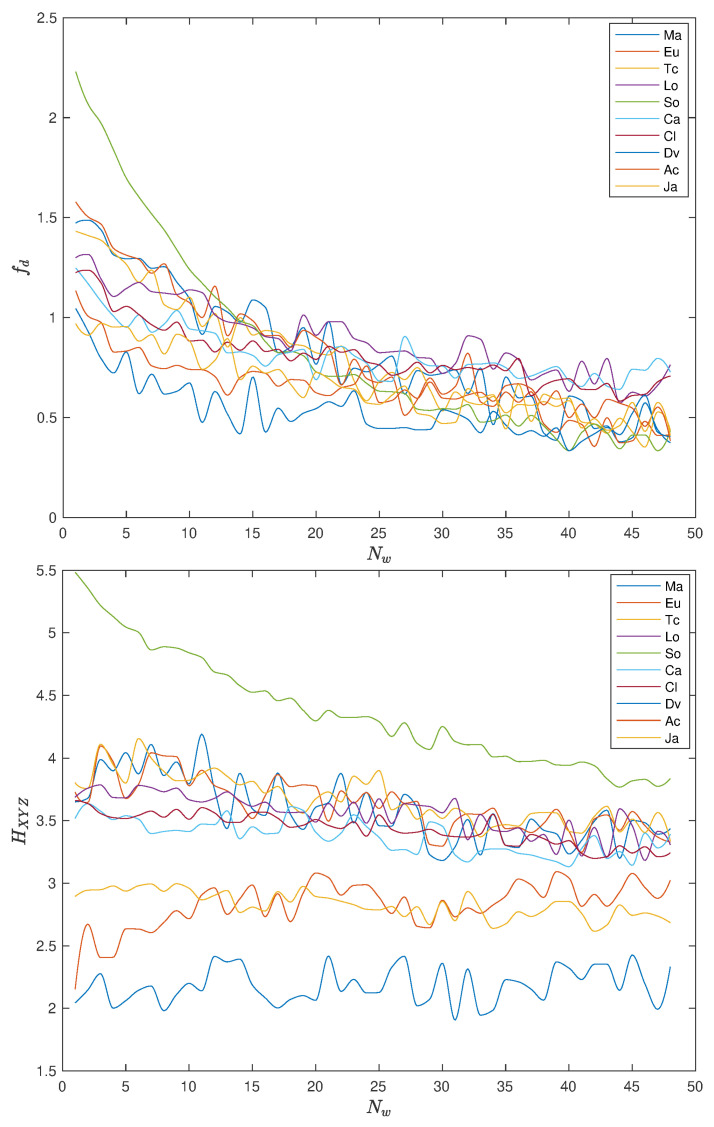
Plot of fractal dimension, $f_{d}$, and Shannon entropy, $H_{XYZ}$, versus $N_{w}$ ($t_{w}\in \left\{5,\ldots ,240\right\}$), with pre-processing $P_{1}$ and using the distances (1a)–(1j).

**Figure 10:**
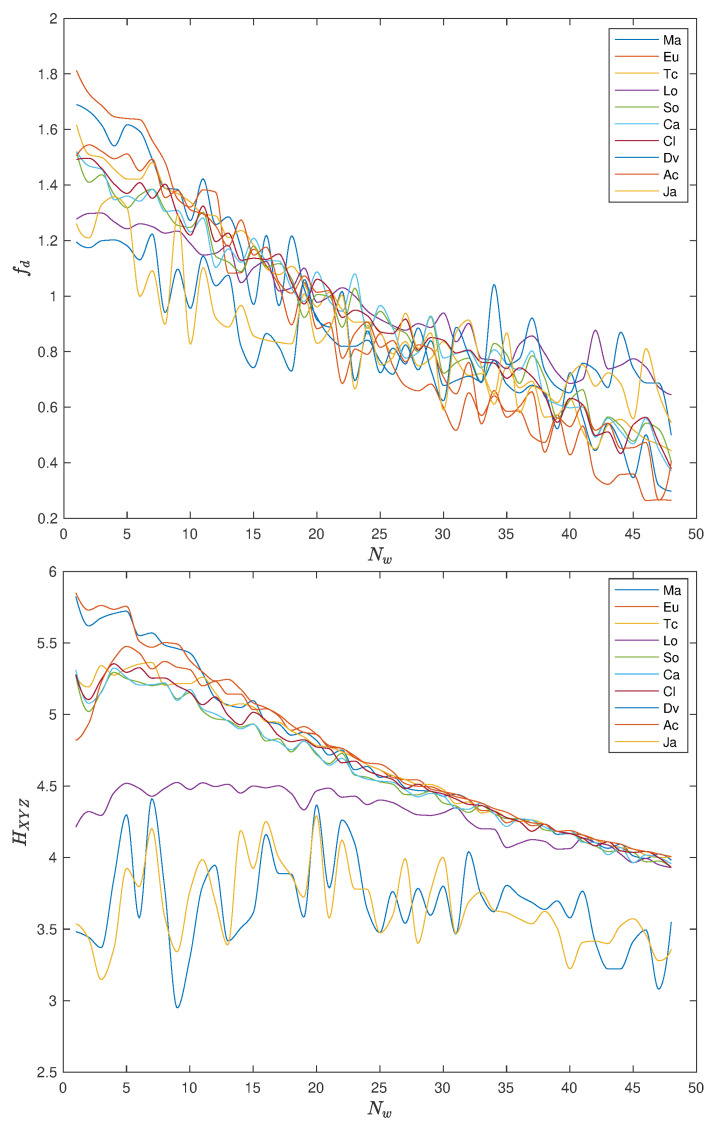
Plot of fractal dimension, $f_{d}$, and Shannon entropy, $H_{XYZ}$, versus $N_{w}$ ($t_{w}\in \left\{5,\ldots ,240\right\}$), with pre-processing $P_{2}$ and using the distances (1a)–(1j). The Manhattan, Euclidean, Tchebychev, Lorentzian, Sørensen, Canberra, Clark, divergence, angular, and Jaccard distances (denoted as {Ma, Eu, Tc, Lo, So, Ca, Cl, Dv, Ac, Ja}).

Figure 9 and Figure 10 show the variation of $f_{d}$ and $H_{XYZ}$ with $t_{w}$, with pre-processing $P_{1}$ and $P_{2}$, respectively, when using the distances (1a)–(1j). For $t_{w}\in \left\{5,\ldots ,240\right\}$, we have correspondingly MDS with $N_{w}$ ($t_{w}\in \left\{3166,\ldots ,65\right\}$) points.

We note some “noise”, but that should be expected due to the numerical nature of the experiments. In general, the two indices decrease with $t_{w}$, revealing, again, the “low pass filtering” effect of the dimension of the time window. We note a considerable difference of the values of $f_{d}$ and $H_{XYZ}$ for small values of $t_{w}$, but a stabilization and some convergence to closer values when $t_{w}$ increases.

**Figure 11:**
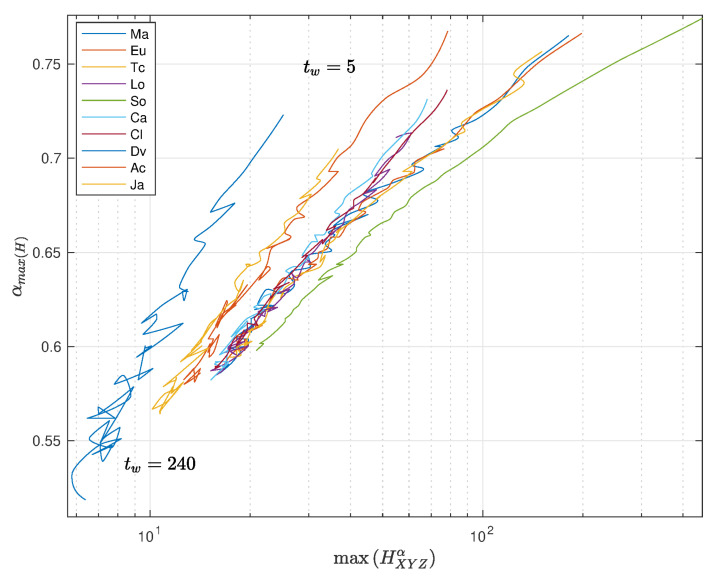
Plot of $\alpha_{max(H)}$ versus $\max\left(H_{XYZ}^{\alpha }\right)$, with $t_{w}\in \left\{5,10,\ldots ,240\right\}$, with pre-processing $P_{1}$ and using the distances (1a)–(1j).

**Figure 12:**
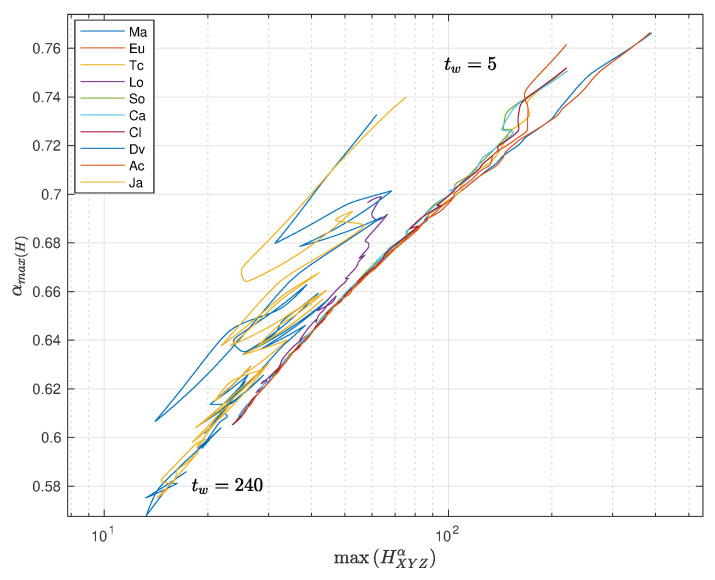
Plot of $\alpha_{max(H)}$ versus $\max\left(H_{XYZ}^{\alpha }\right)$, with $t_{w}\in \left\{5,10,\ldots ,240\right\}$, with pre-processing $P_{2}$ and using the distances (1a)–(1j).

In the case of the fractional entropy, $H_{XYZ}^{\alpha }$, we can tune the value of α to achieve a maximum sensitivity. In other words, we can select the value $\alpha_{max(H)}$ to obtain $\max\left(H_{XYZ}^{\alpha }\right)$. Figure 11 and Figure 12 depict $\max\left(H_{XYZ}^{\alpha }\right)$ vs. $\alpha_{max(H)}$ with $t_{w}\in \left\{5,10,\ldots ,240\right\}$, with pre-processing $P_{1}$ and $P_{2}$, respectively, and using the distances (1a)–(1j).

We verify a strong correlation between the entropy and the value of the fractional order. Furthermore, we note that $0.55\leq \alpha_{max(H)}\leq 0.75$ and $0.57\leq \alpha_{max(H)}\leq 0.77$ for $P_{1}$ and $P_{2}$, respectively, far from integer values and clearly representative of fractional dynamics. For small time windows, each distance has a distinct behavior, but when the time window increases, all distances converge to almost similar points of $\alpha_{max(H)}$, both for $P_{1}$ and $P_{2}$. Obviously, with larger time windows, we have a smaller number of points in the MDS locus, and that influences the result. The convergence towards a common behavior for all distances is observed after the first values of $t_{w}$. This means that we are unraveling the fractional dynamics, that is a characteristic of long-range memory effects embedded in the time series.

For the pre-processing $P_{1}$, the divergence distance produces a slightly separated plot to the left, while for $P_{2}$, we see that position is occupied the divergence and Jaccard distances, but with a fuzzier behavior. As before, we note that the type of pre-processing does not yield any significant modification of the global conclusions.

## 4. Conclusions

Commonly, time is viewed as a continuous and linear flow so that any perturbation, such as noise and volatility, is automatically assigned to the variable under analysis. In other words, since we are entities immersed in the time flow, apparently, we are incapable of distinguishing between perturbations in the time and the measured variable. This paper explored an alternative strategy of reading the relationship between the variables. For that purpose, the DJIA, from 28 December 1959, up to 1 September 2020, was adopted as the vehicle for the numerical experiments. This dataset corresponds to a human-made phenomenon, and therefore, any conjecture about the nature of time is independent of the presently accepted conceptions about its flux. In the proposed approach, the time series was organized into vectors corresponding to specified time windows. Those vectors were then compared by means of a panoply of distances and the resulting information plotted in a three-dimensional space by means of MDS. Indeed, the MDS representation corresponds to a “customized projection” of high-dimensional data into a low-dimensional space. Loosely speaking, we can say “customized projection” since we do not pose any a priori requirements, the algorithm merely being based on the idea of minimizing the difference between the original measurements and the replicated (approximated) value. Therefore, the MDS does not automatically guarantee the success of such a “projection”, but the quality results were assessed by the stress and Shepard diagrams. In the case of the DJIA and the adopted distances, the good quality of the MDS technique was confirmed.

The MDS loci have distinct shapes, according to the type of distance adopted to compare vectors. Therefore, additional tools were necessary to highlight the main characteristics of these representations where time is no longer the explicit variable. For that purpose, several mathematical tools were considered, namely the Shannon entropy and fractal dimension. In all cases, we observed some variability with the time window, which occurs naturally due to the numerical treatment of this type of data. The Shannon entropy and fractal dimension exhibited the same type of behavior, with a progressive variation with the time window and a stabilization toward a common value for large $t_{w}$. While these results can be read merely as the effect of a low pass filtering provided by the large time window, we can also foresee that another property inherent to the DJIA is their origin.

The fractional entropy was brought up to further analyze the MDS locus. This tool allows a better sensitivity to the dataset than the Shannon entropy, since users can tune the calculations by means of the fractional order. In the case of the DJIA, the tuning of α for achieving the maximum entropy revealed not only that such values are independent of the distance, but also that we clearly have orders far from integer values, characteristic of fractional dynamics with non-local effects.

Some concepts are debatable and do not follow the standard orthodoxy, but the set of experiments with an artificial time series allows thinking outside the box and provides a strategy for exploring the texture of time in the perspective of entropy and fractional calculus.
